# A nationwide survey of confidence and knowledge of assessment and management oral conditions amongst a sample of physicians, United Kingdom

**DOI:** 10.1186/s13104-019-4359-0

**Published:** 2019-06-20

**Authors:** Geriatric Medicine Research Collaborative, Carly Welch, Lauren McCluskey, Daisy Wilson, Mary Ni Lochlainn, Joanne K. Taylor, Victoria Gaunt, Kumudhini Giridharan, Benjamin Jelley, Emma Louise Cunningham, Roisin Healy, Mili Doshi

**Affiliations:** University of Birmingham Research Laboratories, Queen Elizabeth Hospital Birmingham, Birmingham, B15 2GW UK

**Keywords:** Mouth care, Education, Older adults

## Abstract

**Objective:**

This study aimed to assess current confidence and knowledge of oral conditions amongst a sample of UK physicians and doctors in training programmes using a web-based survey.

**Results:**

131 survey responses were analysed for doctors from FY1 to consultant grade working within medical specialties. 36.6% and 35.9% of those surveyed expressed that they felt confident diagnosing and managing oral conditions respectively. The median knowledge score was 60%; 65.6% correctly identified the image that demonstrated a squamous cell carcinoma. 91.6% reported that they felt they needed additional training in the diagnosis and management of oral conditions. Neither confidence nor knowledge were affected by grade, specialty, or region of practice.

**Electronic supplementary material:**

The online version of this article (10.1186/s13104-019-4359-0) contains supplementary material, which is available to authorized users.

## Introduction

Oral conditions are prevalent in medical patients, particularly amongst older adults; periodontitis effects up to 80% of older adults [1] and 20–30% are edentulous [1, 2]. Presence of oral conditions correlates with systemic disease, including Chronic Obstructive Pulmonary Disease [3], Chronic Kidney Disease [4] and cardiovascular disease [5], although direction of causality is unknown. Oral conditions have extensive impact upon quality of life, impacting upon psychosocial wellbeing, as well as biological health [6]. However, United Kingdom (UK) undergraduate and postgraduate training includes minimal focus upon oral health assessment and management [7].

Hospitalisation is associated with further oral health deterioration; dental plaque accumulation, gingival inflammation, and mucosal health degradation [8]. In turn, this increases risk of hospital-acquired infections [9] and poor nutrition [10], which can prolong recovery and increase adverse outcomes [11, 12]. Oral assessment is recommended as part of Comprehensive Geriatric Assessment [13]. However, frequency assessments are performed and doctors’ confidence have not been previously examined. This study aimed to evaluate confidence and knowledge of oral health assessment amongst a sample of UK physicians and doctors in training.

## Main text

### Methods

A web-based survey was generated using Google Forms (Additional file 1). Questions included grade of doctor, specialty (for registrars and consultants), region, and previous dentistry study. Respondents were asked the frequency they conducted oral health assessments, and perceived importance of oral health assessment, need for training in oral health assessment, and confidence in diagnosing and managing oral conditions. The second section included five images and short case vignettes; respondents were asked to select the most appropriate answer for each. Images were validated as appropriate examples of pathology, supplied by Mouth Care Matters [14]. A final question asked broadly for additional comments.

The survey was distributed to physicians and doctors in training nationally through social media (Twitter), Royal College of Physicians (RCP) mailing lists, and the Geriatric Medicine Research Collaborative (GeMRC) [15]. When using social media, the survey was not specifically targeted towards any specialty, although this was posted by GeMRC. We specifically targeted geriatricians, considering particular impact of adverse oral health upon older adults. We did not restrict access or dissemination; anyone who had access was able to invite others to participate. We did not collect identifiable information of respondents and were unable to specifically target non-respondents. Survey responses were collected from 1st October 2017 to 1st December 2017. Inclusion criteria were qualified doctors working within the UK of any grade (Foundation Year 1, FY1, to consultant), within a specialty affiliated with the RCP i.e. with general medicine training. The only exclusion criterion was previous dentistry study.

Survey responses were downloaded onto Google sheets and transposed into IBM SPSS Statistics 22 (Chicago, USA). Binary logistic regression analysis was used to assess if region, specialty, or grade of doctor were predictive of frequency of assessment (always vs. others), confidence diagnosis (fairly/very vs. not), confidence managing (fairly/very vs. not) or knowledge (score ≥ 4/5 vs. ≤ 3/5). Specialty was analysed as geriatric medicine compared to others. Grade was analysed as FY1, FY2–CT2, registrars, and consultants. Qualitative data was derived from the final question; presented with direct quotes.

### Results

**Fig. 1:**
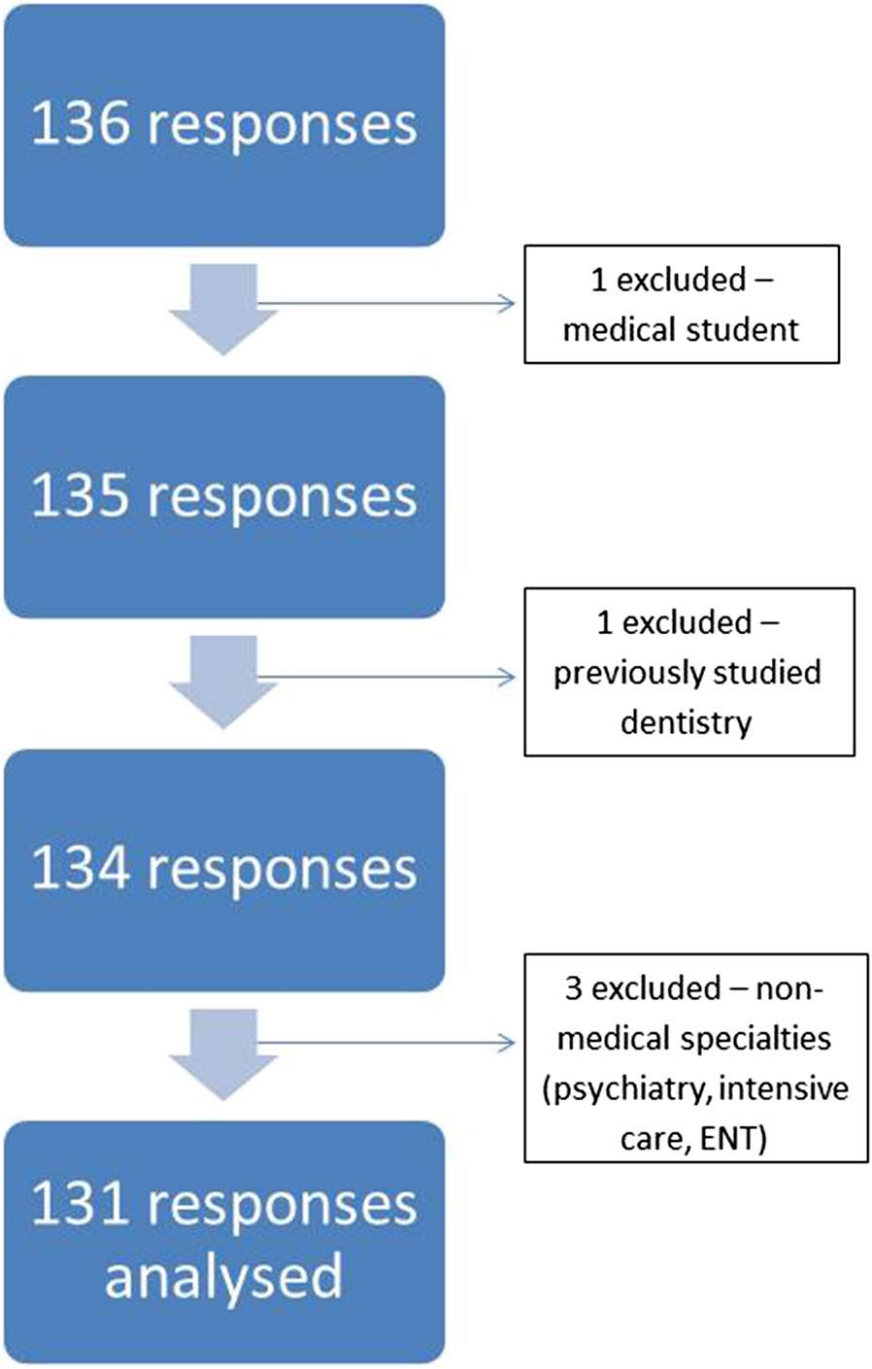
Flowchart showing included and excluded participant responses. 136 responses were initially returned for this survey. A total of five responses were excluded as these were from medical students, a doctor who had previously studied dentistry, and non-medical specialties

136 responses were obtained. Three respondents within specialties not affiliated with the RCP (psychiatry, intensive care, and Ear, Nose, and Throat) were not included. These did not meet our inclusion criteria and were considered non-representative of the sample. One medical student respondent was not included. A respondent who had previously studied dentistry was excluded. Figure 1 depicts included and excluded responses. As of October 2018, there are 30,102 doctors working within general medicine within the UK; our sample represents 0.4% of available respondents [16]. The largest response rate was from geriatricians − 63 (2.7%) out of 2342 [17].

#### Quantitative results

Considering respondents who were included in analysis, 3.1% were FY1 level (first year post-graduation), 11.4% were FY2-CT2 (within 2- to 4 years post-graduation), 71.0% were registrars, and 14.5% were consultants. Considering registrars and consultants, 56.3% were geriatricians. Specialists in alternative medical specialties included gastroenterology, acute medicine, and respiratory medicine. Considering region, 69.5% were from the West Midlands. The summary of respondent demographics is shown in Table 1.

**Table 1:** Demographics of participants who returned responses to our survey

	N	%
Grade		
FY1	4/131	3.1
FY2–CT2	15/131	11.4
Registrar	93/131	71.0
Consultant	19/131	14.5
Specialty (registrars and consultants only)		
Geriatric medicine	63/112	56.3
Other medicine	49/112	43.8
Region		
West Midlands	91/131	69.5
London	7/131	5.3
North West of England	9/131	6.9
East Midlands	2/131	1.5
Severn	1/131	0.8
Kent, Surrey, and Sussex	1/131	0.8
Wales	13/131	9.9
Scotland	5/131	3.8
Northern Ireland	2/131	1.5

71% were registrars, 56.3% were specialising in geriatric medicine, and 69.5% were working within the West Midlands

Overall, 21.4% expressed they always looked inside patients’ mouths during routine assessment; a further 65.6% expressed they sometimes looked inside patients’ mouths. Nearly all, 96.9%, considered it important to look in patients’ mouths as part of assessment, but only 36.6% felt confident in diagnosing oral conditions. Additionally, 64.1% expressed they did not feel confident managing oral conditions. The majority, 70.2%, rated it high importance (7 or greater on a Likert scale of 1 to 10) for doctors to have oral health training. However, only 8.4% felt they had received sufficient training to diagnose oral conditions, and 91.6% felt they needed additional training in diagnosis or management of oral conditions.

The five images and associated case vignettes demonstrated dental plaque, xerostomia, stomatitis, aphthous ulceration, and tongue squamous cell carcinoma. The median score was 60% (IQR 60–80%); three out of five correct answers. The aphthous ulcer was correctly identified by 93.1%, whereas 44.3% identified stomatitis. Dental plaque was identified by 65.6% and xerostomia by 51.9%. Importantly, 65.6% correctly identified the tongue squamous cell carcinoma. Other answers given included salivary gland carcinoma (11.4%), lymphoma (0.8%), melanoma (0.8%), plaque (0.8%), and “Don’t know” (20.6%). Quantitative results are shown in Table 2.

**Table 2:** Quantitative results of survey responses including percentages with 95% confidence intervals

	N (Total = 131)	%	95% CI
Frequency of mouth care assessment			
Never	2	1.5	0.4–5.4
Rarely	15	11.5	7.1–18.0
Sometimes	86	65.6	57.2–73.2
Always	28	21.4	15.2–29.2
Perceived importance of mouth care training			
Score 1–3	5	3.8	1.6–8.6
Score 4–6	34	26.0	19.2–34.1
Score 7–10	92	70.2	61.9–77.4
Perceived importance of mouth care assessment (%yes)	127	96.9	92.4–98.8
Confidence diagnosing oral conditions			
Not	83	63.4	54.8–71.1
Fairly	47	35.9	28.2–44.4
Very	1	0.8	0.1–4.2
Confidence managing oral conditions			
Not	84	64.1	55.6–71.8
Fairly	46	35.1	27.5–43.6
Very	1	0.8	0.1–4.2
Perceived sufficient training (%yes)	11	8.4	4.7–14.4
Perceived need for further training (%yes)	120	91.6	85.6–95.2

	Median	IQR
Knowledge score	60% (3/5)	60–80

21.4% (15.2–29.2%) of respondents stated that they always performed mouth care assessment as part of a routine clinical assessment; whereas 96.9% (92.4–98.8%) expressed that they perceived mouth care assessment to be important

Logistic regression analysis did not reveal any difference in confidence, frequency of assessment, or knowledge of oral conditions between different grades of physicians, different specialties, or different regions (Additional file 2).

#### Qualitative results

A recurrent theme in responses was that they felt comfortable assessing for common oral health conditions (e.g. candida infection) but did not feel they had either expertise or experience to identify less common pathology.

*“I look for a few things that appear commonly in my area of practice. Beyond that I would send the patient to the dentist or max*-*fax clinic”*

*“Confident with simple oral issues such as thrush but not with other issues such as ulcerative diagnoses”*

Respondents appreciated importance of oral health assessment in clinical practice and frequently suggested further training was required.

*“Need to start more focus in med school and foundation year curriculum and build on this rather than just providing lectures/educational resources”*

*“Solving poor oral care will undoubtedly improve patient nutritional intake and reduce aspiration risk. It is also our responsibility to highlight the importance to our allied healthcare professional colleagues”*

*“Oral care poorly understood and terrible diagnosis rates—needs a lot more attention!”*

It was suggested that blaming inadequate training was overly simplistic and that wider cultural and systematic factors contribute to poor oral health assessment within clinical practice. It was suggested that in order to improve undergraduate and postgraduate education of mouth care assessment and management, consultants take responsibility for improving their own knowledge and skills. It was also suggested that access to a hospital dentist may have positive impacts upon patient outcomes.

*“We all have a responsibility to ensure we are up to date and are equipped with the best clinical and evidence*-*based knowledge. I think it is all too easy to blame training. There is nothing stopping trainees or consultants from attending dental/oral medicine clinics if they feel this is something that they are weak on”*

*“Good access to in hospital dentist arguably more appropriate than training us to treat things outside our capability*—*we don’t have kit or training to do more than very basics”*

### Discussion

Within our sample of UK physicians, a significant proportion did not feel confident assessing, diagnosing, or managing oral conditions. This was demonstrated across all grades from FY1 to consultant. The median test score was 60%, which suggests not only deficiencies in confidence, but also deficiencies in competence. The majority were able to correctly identify aphthous ulceration, whereas correct identification of other pathology was lower. This corroborates with our qualitative results, whereby respondents expressed they felt confident identifying common oral conditions, but less confident assessing and managing rarer, more complex diagnoses. Of greatest concern, only 65.6% correctly identified the tongue squamous cell carcinoma. However, a further 13.7% considered the figure showed another malignant diagnosis. In practice, this should have led on to referral to other specialties for further investigation and management in 79.3% of cases. Only one person considered the figure showed plaque, the only benign option for this question. The remainder expressed they did not know what the image showed instead of attempting an answer.

Although older adults may be most vulnerable to adverse effects of poor mouth care [18, 19], mouth care assessment remains a vital part of routine assessment for all age groups [20]. Within our sample, doctors did not feel confident in performing oral health assessment and requested further training. Frequency of assessment, confidence in diagnosing or managing oral conditions, and knowledge were not affected by grade, specialty, or region within the UK. We, therefore, consider this may be a widespread problem. A larger sample would be informative in assessing if there are associations between these factors, which our sample may have been under-powered to detect. Further formal evaluation of confidence and competence of mouth care assessment and management amongst UK physicians should be driven nationally to assess trends at scale. Including surgical specialties in a larger sample would be beneficial, in determining how physicians compare. This knowledge could potentially be used to determine need for restructuring postgraduate training with focus upon oral health.

We consider restructuring of training with early focus within undergraduate training that is reinforced throughout postgraduate training may be beneficial. Concurrently, postgraduate education should focus on training consultants and senior registrars so they can continue to maintain their own skills and contribute to training of junior colleagues. One way this may be achieved is through inter-disciplinary teaching and closer collaborative working with dentists, with potential to dramatically improve patient care and holistic management. At present, few hospitals within the UK have an inpatient dentistry service. Dental procedures are often required to be delayed until the patient is discharged into the community. Considering adverse health outcomes, including negative effects on nutrition [10], social engagement [21], and systemic inflammatory response [3–5], this may prolong recovery. In addition, older adults infrequently attend dental appointments [22]; frail and/or cognitively impaired older adults in care homes particularly struggle to access community dental care [23]. Closer collaborative working with dentists within secondary care may enable patients to access expertise when necessary, but also help to improve doctors’ knowledge and skills.

We suggest that a nationwide programme of education for doctors of all grades may be beneficial. This should include targeted local interventions, as well as innovative national quality improvement strategies using webinars, social media, and public engagement with older adults. The Mouth Care Matters programme focusses on improving oral health through training and educating healthcare professionals and has been successfully introduced into UK selected hospital sites with promising results [24]. Analysis of the effects of this programme upon patient-related outcomes will be vital in demonstrating need for nationwide implementation to stakeholders.

## Limitations

- It is not possible to be certain if responders who identified the squamous cell carcinoma would have made a referral in practice or if this relates to bias of multiple-choice questions.
- All images presented were two-dimensional. In practice, physicians would be able to examine pathology in situ, which would include evaluating firmness and associated odour.
- We acknowledge that our sample was small compared to overall number of physicians working within the UK, and may not be representative. We cannot rule out participation bias; doctors who were more engaged in importance of oral health assessment, or more aware of their own deficiencies may have been more likely to complete the survey. However, no incentives were provided for survey completion that would have caused a conflict of interest. Survey participation was fully voluntary and was not specifically targeted towards doctors based on prior knowledge of confidence or competence in oral health assessment.
- It is not possible to know the total number of doctors who were aware of the survey, due to dissemination methods. It is probable that this was far lower than the total number of physicians within the UK, and thus, the true response rate may have been higher.
- We did not collect details of undergraduate training (e.g. country of training), which may have affected results.

## Additional files

**Additional file 1.** Blank version of original survey used. The file includes the original survey that was disseminated to all respondents who participated.

**Additional file 2.** Results of logistic regression analysis for frequency of assessment, confidence, and knowledge. Table showing results of logistic regression analysis for the effect of region, specialty, and grade upon likelihood of frequently assessing oral health, respondents feeling confident in diagnosing and managing oral health conditions, and scoring 4 or more on the knowledge quiz. No results were significant at group level, therefore, individual odds ratios are not shown.

## Data Availability

The datasets used and analysed during the current study are available from the corresponding author on reasonable request.

## Abbreviations

FY1: Foundation Year 1 Doctor
FY2: Foundation Year 2 Doctor
IQR: interquartile range
UK: United Kingdom
