# Supplementary material for: A nationwide survey of confidence and knowledge of assessment and management oral conditions amongst a sample of physicians, United Kingdom
Source: BMC Res Notes. 2019 Jun 20;12:348. doi: 10.1186/s13104-019-4359-0 (PMC6585010; doi:10.1186/s13104-019-4359-0)
Supplement: Supplementary file 1 — Additional file 1. Blank version of original survey used. The file includes the original survey that was disseminated to all respondents who participated. [file 13104_2019_4359_MOESM1_ESM.pdf]

Mouth care educational survey

Survey of knowledge and current practices relating to mouth care by doctors in within the UK

* Required

1. **What region do you work in?** *

Mark only one oval.

- Scotland
- Northern Ireland
- North East England
- North West England
- Yorkshire
- West Midlands
- East Midlands
- Severn
- Peninsula
- Wales
- Wessex
- Oxford
- London (all)
- Kent, surrey or sussex
- East of England
- Other: ______________________

2. **What is your grade?** *

Mark only one oval.

- FY1 *Skip to question 4.*
- FY2 *Skip to question 4.*
- Core Medical Training *Skip to question 4.*
- GPVTS *Skip to question 4.*
- Core Surgical Training *Skip to question 4.*
- Medical StR *Skip to question 3.*
- Surgical StR *Skip to question 3.*
- Consultant *Skip to question 3.*
- Other: ______________________ *Skip to question 3.*

Specialty

3. **What is your main specialty?** *

____

Mouth care educational survey

Survey of knowledge and current practices relating to mouth care by doctors in within the UK

4. **On a scale of 1 to 10 how important do you think it is for doctors to have oral health training (1 being the least importance, 10 being very important)** *
Mark only one oval.

1	2	3	4	5	6	7	8	9	10
◯	◯	◯	◯	◯	◯	◯	◯	◯	◯

5. **Have you previously studied dentistry?** *
Mark only one oval.
◯ Yes
◯ No

6. **Do you look in patients' mouths as part of routine assessment?** *
Mark only one oval.
◯ Always
◯ Sometimes
◯ Rarely
◯ Never

7. **5. Do you think it is important to look into patients' mouths as part of assessment?** *
Mark only one oval.
◯ Yes
◯ No

8. **6. How confident do you feel diagnosing oral conditions?** *
Mark only one oval.
◯ Very Confident
◯ Fairly Confident
◯ Not Confident

9. **How confident do you feel managing oral conditions?** *
Mark only one oval.
◯ Very Confident
◯ Fairly Confident
◯ Not Confident

10. **Do you feel that you have received sufficient training to diagnose oral conditions?** *
Mark only one oval.
◯ Yes
◯ No

11. **Do you feel you need additional training in the diagnosis or management of oral conditions?** *

Mark only one oval.

- Yes
- No

12. **Any other comments?**

Mouth care knowledge survey

Survey of knowledge of abnormal mouth pathology

30 year old patient with learning disabilities, has been in hospital with pneumonia for 10 days

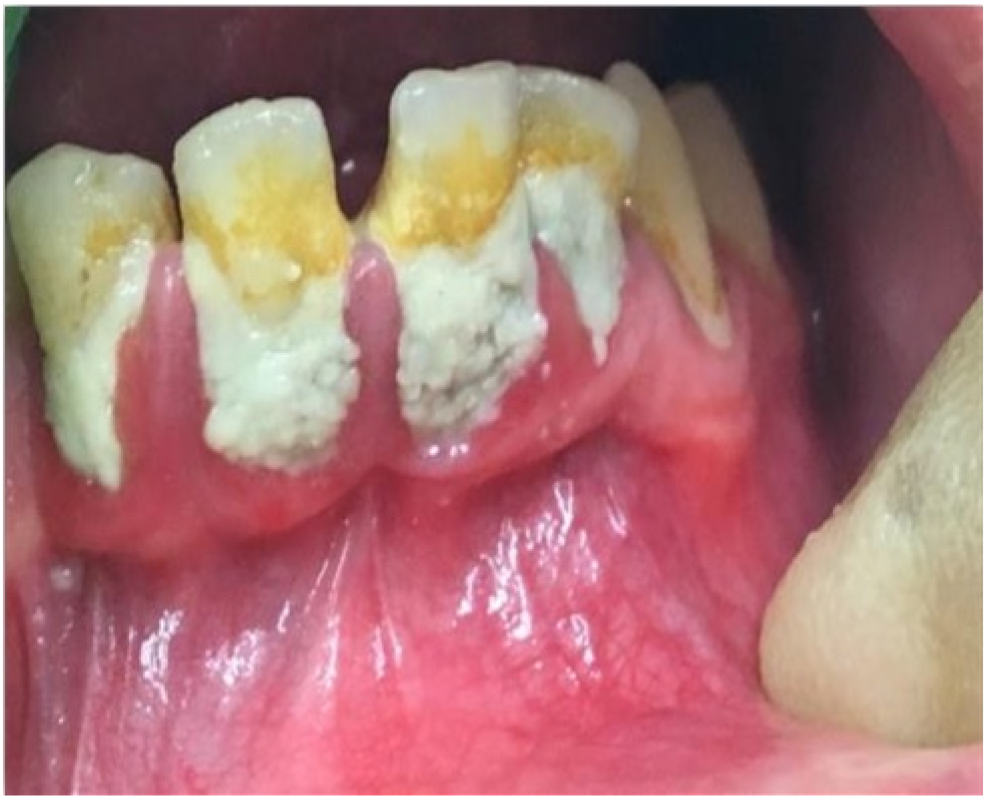

13. **Does this image show:** *

Mark only one oval.

- Oral thrush
- Dental calculus
- Oral cancer
- Noma
- Don't know

A 70 year old female on a cardiac ward complaining of a sore tongue

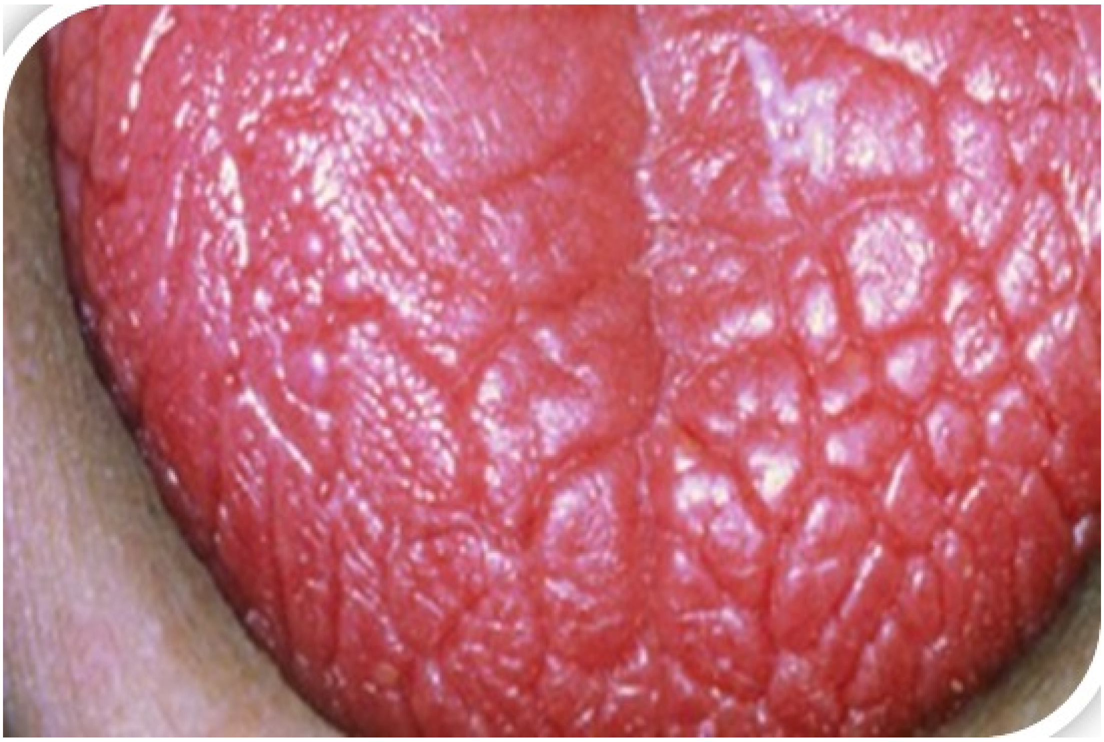

14. **Does this image show:** *

Mark only one oval.

- Xerostomia
- Oral cancer
- Noma
- Macroglossia
- Candidiasis
- Don't know

80 year old female. On removal of an upper denture her palate has the following appearance

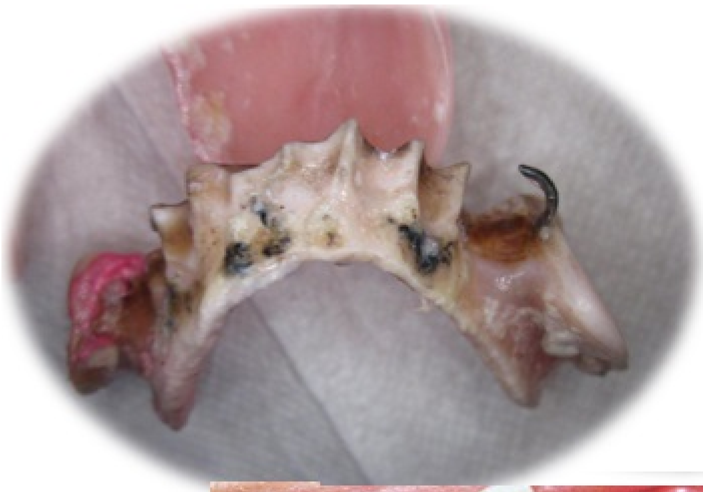

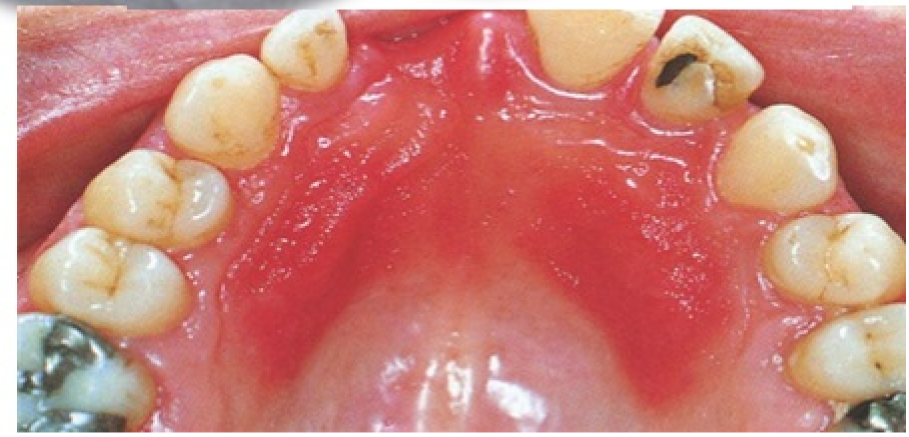

15. **Does this show:** *
Mark only one oval.

- ◯ Oral Cancer
- ◯ Dental caries
- ◯ Normal (for dentures)
- ◯ Stomatitis
- ◯ Candidiasis

70 year old post surgery patient with a sore mouth

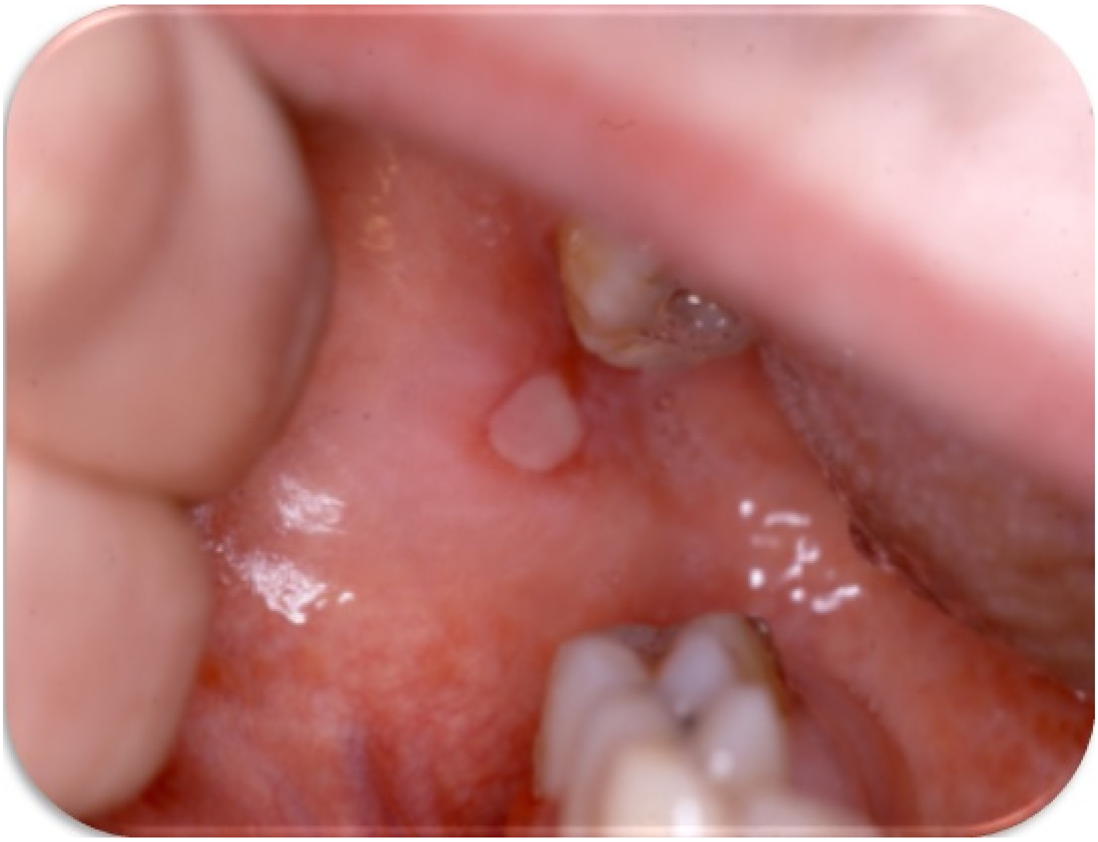

16. **Does this show:** *

Mark only one oval.

- Apthus ulcer
- Candidiasis
- Oral cancer
- HPV infection
- Don't know

67 year old male with a sore tongue admitted after a fall

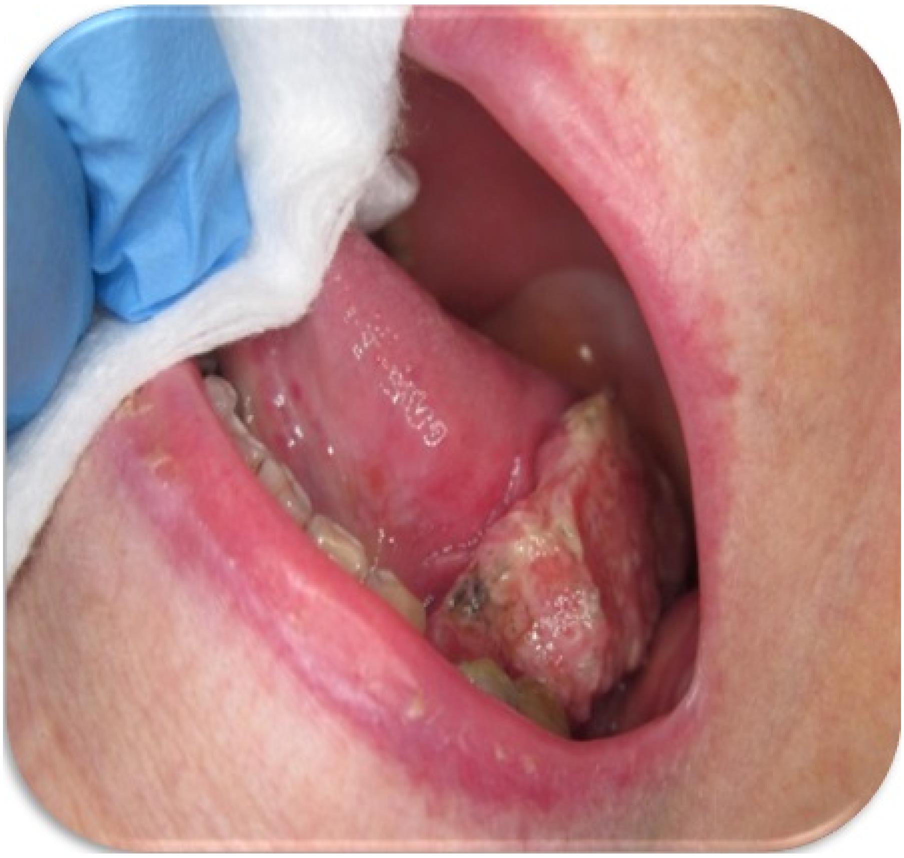

17. **Does this show:** *

Mark only one oval.

- Plaque
- Lymphoma
- Tongue small cell carcinoma
- Salivary gland carcinoma
- Melanoma
- Don't know

Thank you for completing our survey!

Powered by
Google Forms
