# Supplementary material for: A nationwide survey of confidence and knowledge of assessment and management oral conditions amongst a sample of physicians, United Kingdom
Source: BMC Res Notes. 2019 Jun 20;12:348. doi: 10.1186/s13104-019-4359-0 (PMC6585010; doi:10.1186/s13104-019-4359-0)
Supplement: Supplementary file 2 — Additional file 2. Results of logistic regression analysis for frequency of assessment, confidence, and knowledge. Table showing results of logistic regression analysis for the effect of region, specialty, and grade upon likelihood of frequently assessing oral health, respondents feeling confident in diagnosing and managing oral health conditions, and scoring 4 or more on the knowledge quiz. No results were significant at group level, therefore, individual odds ratios are not shown. [file 13104_2019_4359_MOESM2_ESM.docx]

Table S1 - Results of logistic regression analysis for frequency of assessment, confidence, and knowledge.

There was no effect of region, specialty, or grade upon likelihood of frequently assessing oral health, respondents feeling confident in diagnosing and managing oral health conditions, and scoring 4 or more on the knowledge quiz. No results were significant at group level; therefore, individual odds ratios are not shown.

|  | | Wald | Freedom | p |
| --- | --- | --- | --- | --- |
| Frequency of assessment (Always) | Region | 2.64 | 8 | 0.96 |
|  | Specialty | 0.07 | 1 | 0.79 |
|  | Grade | 0.72 | 1 | 0.40 |
| Confidence diagnosing (Fairly or very) | Region | 5.44 | 8 | 0.71 |
|  | Specialty | 2.58 | 1 | 0.11 |
|  | Grade | 0.02 | 1 | 0.90 |
| Confidence managing (Fairly or very) | Region | 6.09 | 8 | 0.64 |
|  | Specialty | 0.90 | 1 | 0.34 |
|  | Grade | 1.75 | 1 | 0.19 |
| Knowledge (score ≥ 4/5) | Region | 0.22 | 8 | 1.00 |
|  | Specialty | 0.19 | 1 | 0.67 |
|  | Grade | 2.68 | 1 | 0.10 |
